# Pulmonary aspergillosis: a challenging condition in the whole world

**DOI:** 10.4322/acr.2020.240

**Published:** 2021-01-28

**Authors:** Vitorino Modesto dos Santos

**Affiliations:** 1 Hospital das Forças Armadas, Departamento de Medicina, Brasília, DF, Brasil; 2 Universidade Católica de Brasília, Brasília, DF, Brasil

**Keywords:** Aspergillosis, Autopsy, Diagnosis

Dear Editor,

We read the report of invasive aspergillosis in a 38-year-old male diagnosed with the Hodgkin lymphoma refractory to chemotherapy and autologous transplantation. Three months after the allogeneic HLA-matched stem cell transplantation, he evolved with infections by *Clostridioides* (*Clostridium*) *difficile*, adenovirus, and *Aspergillus*.^1^ The bacterial infection was treated during life, while both adenoviral enteritis and hepatitis and the severe invasive pulmonary aspergillosis had a postmortem diagnosis. The authors made clear a wide spectrum of complications related to Hodgkin lymphoma management as a whole and emphasized the role of the hidden opportunistic infections. They highlighted the limitations of current diagnostic tools for aspergillosis, including cultures in blood and aspirates, tissue sampling, as well as serum galactomannan test.^1^ The patient underwent the best care of modern medicine in a high-income country. However, worthy of note was the final seven days of a downhill clinical course, despite utilizing three antifungal medications - caspofungin, voriconazole, and posaconazole.^1^

In this setting, comments about diagnostic challenges seem appropriate in three Brazilian patients with pulmonary aspergillosis; two also had lung adenocarcinomas.^2^^-^^4^ These 76-year-old and 64-year-old males with right lung malignancies underwent a schedule of pemetrexed disodium, cisplatin, and dexamethasone for near one year. The management with voriconazole in both cases resulted in successful outcomes.^2^^,^^3^ The first one had accentuated lymphopenia, and chest imaging showed a cavity in a left lung nodule mimicking central necrosis within an implant of the lung adenocarcinoma, but qualitative PCR analysis and cultures obtained in aspirates revealed *Aspergillus*.^2^ The second with neutropenia lower than 500/mm3 had the diagnosis of aspergillosis by histopathology and culture of biopsy samples, as well as positive galactomannan test.^3^ The main initial concern, in this case, was about misdiagnosis involving most common agents of pneumonia and sepsis associated with febrile neutropenia in malignancies.^3^

The last case, but not the least, refers to a 76-year-old immunocompetent woman with bilateral bronchiectasis, multiple nodules, and a tree-in-bud image in the lung base.^4^ During the last four months, she utilized moxifloxacin, amoxicillin plus clavulanate, moxifloxacin, piperacillin, and tazobactam without significant clinical improvement. The major concerns, in this case, were pulmonary tuberculosis and scar cell carcinoma. Complementary exams showed neutrophilia, positive galactomannan test (0.7 index), and negative cultures for fungi in samples of the bronchoalveolar aspirate. Furthermore, the repeated galactomannan test was negative after the itraconazole administration, and the chest images of control revealed a decrease in the diameters of pulmonary nodules. The final diagnosis was chronic pulmonary aspergillosis responsive to itraconazole.^4^

The growing population pertaining to the oldest age groups, in addition to the increased number of patients submitted to organ transplantations, chemotherapy, and other immunosuppressive treatments, are predisposing to opportunist fungal infections. Due to nonspecific manifestations, these conditions are frequently underdiagnosed, misdiagnosed, or neglected and often related to late diagnosis and very poor outcomes. The current low rate of postmortem studies may cast doubt on the estimated prevalence of these infections, especially in low-income regions with limited diagnostic resources.
